# From plant genetic resources to cosmetic active ingredients: when science meets regulation and market rules

**DOI:** 10.12688/openreseurope.20113.1

**Published:** 2025-06-18

**Authors:** F. Bourgaud, R. M. Twyman, K.-M. Oksman-Caldentey

**Affiliations:** 1 Université de Lorraine, CNRS, LRGP, F-54000, Nancy, France; 2 TRM Ltd, Scarborough, UK; 3 VTT Technical Research Centre of Finland Ltd, Espoo, Finland

**Keywords:** Cosmetics, sustainability, efficacy, plant-based resources, regulatory framework, biotechnology

## Abstract

The use of plants as a source of cosmetic ingredients has a long history, but has become increasingly important over the last three decades as consumers become more aware of the provenance of cosmetic products and their impact on the environment. Modern cosmetic ingredients must not only be safe, effective and sustainable, but must also comply with a complex framework of regulations, only some of which are internationally standardized. Consumers are also presented with an overlapping and poorly defined set of certification schemes offering claims of sustainability and environmental benefits. In this brief review article, we look at the regulations governing plant-based ingredients in the cosmetics industry, and how these intersect with modern biotechnological approaches for the development of ingredients with proven efficacy and sustainability. We showcase the example of *InnCoCells*, the first Horizon project dedicated to the development of cosmetic ingredients based on sustainable plant-based resources.

## Plan language summary

Many of our cosmetic ingredients are sourced from plants, which provide a natural reservoir of bioactive compounds that improve skin health. However, the development of plant-based ingredients in hindered by conflicting international regulations and certification schemes that cover access to genetic resources, permitted substances, and evidence of safety, efficacy and environmental sustainability. An important aspect is the role of biotechnology in cosmetics because biotechnology can make it easier to produce cosmetic ingredients in a sustainable manner from precious plant-based resources. The InnCoCells project, the first Horizon project completely dedicated to the development of natural cosmetic ingredients with proven scientific efficacy, is helping to raise awareness about these issues.

## Disclaimer

The views expressed in this article are those of the author(s). Publication in Open Research Europe does not imply endorsement of the European Commission.

## Introduction

The global cosmetics market was valued at US$424.72 billion in 2024 and is predicted to reach US$760.61 billion by 2034, representing a compound annual growth rate of 6% ( Precedence Research, 2024). Current trends in cosmetics are mainly related to increasing consumer demands for validated efficacy, environmental sustainability, and the removal of synthetic chemicals and their potential for harm. Consumers see a strong link between health and beauty, so they desire safe, effective and personalized products that promote healthy aging. This has created a demand for natural cosmetic products, and manufacturers such as L’Oréal, Procter & Gamble, Patanjali Ayurved, and Estee Lauder are continuously engaged in the production of new herbal and natural ingredients to satisfy this growing market. Plant extracts and plant-derived ingredients will therefore continue to be important raw materials for the future of the cosmetics industry.

## Plants as a source of cosmetic ingredients

The prominent role of plant-based ingredients in today’s cosmetic products is not a modern paradigm. Indeed, plants have been used as a source of cosmetic ingredients since antiquity ( McMullen & Dell’Acqua, 2023). However, the extensive use of plant extracts in beauty products expanded in the early 1990s, reflecting a rapid shift away from the use of raw animal-based products due to risks associated with bovine spongiform encephalopathy and related diseases ( Brown, 1997). This has coincided with a more gradual trend towards greater sustainability and the replacement of synthetic, oil-derived chemical ingredients such as preservatives with natural alternatives.

To understand why plants are so popular in human cosmetic products, we should consider that terrestrial plants have been on earth for more than 450 million years ( Morris *et al.,* 2018), and have adapted to tolerate oxidative stress brought about by harsh environmental conditions ( Sperling *et al.,* 2022), as well as interactions with pathogens, parasites and insect pests ( Kapoor *et al.,* 2023). Plants have evolved defense systems ( Rieseberg *et al.,* 2023) including metabolic responses that buffer oxidative bursts, hinder pathogens ( Ramaroson *et al.,* 2022) and deter infestations with insects ( Kortbeek *et al.,* 2019). Today’s plants therefore provide a rich source of so-called secondary metabolites, which are not essential for plant growth and development but confer important ecological advantages due to their specific bioactivities. Many such metabolites are currently approved as drugs in the pharmaceutical industry because of their strong and specific pharmacological properties ( Miralpeix *et al.,* 2013). The chemical diversity of plants is much higher than any chemical library made by humans and thus the plant kingdom represents an enormous reservoir of bioactive molecules to be discovered – not only for pharmaceutical applications but also the cosmetics sector. Some of these metabolites are widely distributed, such as phenolics ( Kähkönen *et al.,* 1999) and carotenoids ( Young & Lowe, 2001), both of which act as antioxidants. Others are only found in certain plant families or species, such as particular alkaloids and terpenoids ( Oksman-Caldentey & Inzé, 2004). These molecules are useful to humans because pathophysiological processes in animals and plants are related. For example, inflammation and aging-related effects in humans are associated with oxidative damage ( Leonarduzzi *et al.,* 2012) which are inhibited by treatment with antioxidant and anti-inflammatory compounds produced in plants ( Im, 2020; Phan *et al.,* 2018). Plant extracts are therefore valued for their anti-aging effects in cosmetic products ( Zaid & Al Ramahi, 2019; Zhu *et al.,* 2022). Other valuable plant-derived secondary metabolites confer resistance to pathogens. Although plant pathogens generally do not infect animals (and vice versa), plant metabolites that inhibit the growth of plant pathogens can also inhibit other microbes, making them suitable as natural preservatives, which are highly sought after by the cosmetic industry ( Bouarab Chibane *et al.,* 2019).

Although plants are popular materials for cosmetics, it takes more than science and technology to transform plant extracts into valuable cosmetic products. Indeed, the development of cosmetic active ingredients requires compliance with multiple international regulations, and our intention in this article is to describe the major regulatory challenges that a plant extract must successfully overcome to meet the expectations of the cosmetic market.

## Use of plant genetic resources

Plants are part of the world’s terrestrial biological diversity. Therefore, the use of any plant species must be considered in terms of the international regulations defining access to genetic resources. The Rio Convention on Biological Diversity ( Secretariat of the Convention on Biological Diversity, 2023) recognizes the right of countries to control access to their own genetic resources ( Secretariat of the Convention on Biological Diversity, 2023) and has been ratified by 196 countries. The Rio Protocol was supplemented by the Nagoya Protocol (2010) on access to genetic resources and the sharing of benefits arising from their use ( Secretariat of the Convention on Biological Diversity, 2015). It entered into force on 12 ^th^ October 2014. The Nagoya Protocol establishes fundamental obligations for contracting countries to take measures surrounding access to genetic resources, benefit sharing, and compliance.

*Access obligations* require that any institution, such as a cosmetics company or research laboratory working on the development of plant-based products, takes measures at the national level ensuring that it:

-   creates legal certainty, clarity, and transparency

-   establishes clear rules and procedures for preliminary informed consent and mutually agreed terms

-   can provide a permit or equivalent to demonstrate that access is granted

-   creates conditions to promote and encourage research contributing to the conservation and sustainable use of biodiversity

*Benefit-sharing obligations* require that the relying party offers an equitable share of the benefits arising from the use of the genetic resources with the contracting party providing the genetic resources through mutual agreement. This includes research and development on the genetic or biochemical composition of genetic resources, in addition to applications and commercialization. Benefit sharing can be monetary, in the form of a deposit and/or royalties, or non-monetary such as sharing research results, or promoting businesses and economies from which the genetic resources originate.

*Compliance obligations* imply that the party utilizing genetic resources will support compliance with national laws of the party providing genetic resources. In particular, the relying party must take measures to monitor the use of genetic resources once they have left a country by carefully following effective control points at each stage of the value chain (research, development, innovation, marketing, and commercialization). In practical terms, the party wishing to access genetic resources must first consider the country of origin and comply with both its own national regulations but also the regulations in force in the country holding the genetic resources. National regulations are extremely diverse. For example, a company in France wishing to use local genetic resources for research only must request a permit from the Ministry of Ecological Transition, whereas no research permit is necessary in Germany.

## Approved lists of plant species and extracts for cosmetic products

Many plants produce toxic compounds, so both the plant species and the technological processes used to extract and prepare ingredients must be approved before inclusion in a cosmetic product. Official documents regulating plant authorizations are issued by countries based on risk assessments. For example, China has a list of permitted plant extracts found in the Inventory of Existing Cosmetic Ingredients in China (IECIC), the most recent version of which was published in 2021 by the Chinese National Medical Product Administration ( NMPA, 2021). Initially, the IECIC list contained 8972 already used ingredients, including extracts defined by the plant species and often a particular organ, such as roots or flowers. These ingredients were considered approved in China as long as historical maximum concentrations were not exceeded (these maximum values were provided for each listed ingredient). For example, *Scutellaria baicalensis* Georgi extract has three entries in IECIC2021: root powder (no. 03075), root extract (no. 03076), and plant extract (no. 03077), the latter meaning that any part of the plant may be used. Conversely, *Scutellaria lateriflora* L. is not listed in IECIC2021, even though it is widely used in herbal medicine ( Awad *et al.,* 2003).

Plant extracts that are not listed in IECIC2021 are considered new cosmetic ingredients (NCIs) and require registration with NMPA (2021) before they can be used as ingredients for cosmetics sold in China. Depending on the level of risk represented by an NCI, this registration process will follow either a high-risk or low-risk route. The high-risk track includes a mandatory toxicology report demonstrating product safety, which may sometimes require animal testing at the request of the Chinese authorities. The low-risk track involves the completion of online dossiers. In this case, the NCI is considered to be notified and can be used to manufacture a new cosmetic product in China and/or import it. Only six NCIs were notified in 2021, but this increased to 41 in 2022, 68 in 2023, 101 in 2024, and 11 more as of February 2025, showing that companies producing cosmetic ingredients are becoming more accustomed to China's regulatory guidelines. Animal testing for cosmetic ingredients has been banned in the European Union since 11 ^th^ March 2009 ( European Commission, 2009). Therefore, any company wishing to introduce a high-risk NCI would have to choose between the Chinese and European markets. Given these conflicting regulatory issues, we are unaware of any European cosmetic company having filed for the registration of a high-risk NCI in China.

## New trends in green cosmetics

The trend in consumer demand for more sustainable, eco-friendly cosmetic products mirrors the general growth in the market for natural ingredients. From a technological perspective, this demand translates into the application of green chemistry principles during ingredient manufacturing ( Anastas & Eghbali, 2010). However, as appealing as it may be to the public, the term “green” is extremely vague. The lack of a common definition makes it difficult to distinguish genuinely sustainable products from “greenwashing”. To overcome this issue, various certification systems have emerged to verify product qualities throughout the manufacturing chain, including the supply of genetic resources used as raw materials, ingredients, production processes, transportation and storage, packaging, and waste management. Other standards involve life cycle analysis methods that assess aspects such as eco-responsibility through the measurement of energy and water use and/or carbon emissions ( Rocca *et al.,* 2023). For example, the COSMOS standard was created by five certification agencies in Europe: Bundesverband der Industrie- und Handelsunternehmen ( BDIH, 2023), Cosmebio ( Cosmebio, 2023), Ecocert ( Ecocert, 2023), ICEA ( ICEA, 2023) and the Soil Association ( Soil Association, 2023). Many additional certification standards have been developed over the last decade such as the Union for Ethical Biotrade (UEBT), NATRUE, Rainforest Alliance, Responsible Sourcing Palm Oil (RSPO), and Responsible Mica Initiative (RMI).

Biotechnology plays an increasingly prevalent role in the manufacture of plant-based ingredients so it is also important for the public to understand how this aligns with the certification programs. Concerning the use of genetically modified organisms (GMOs), the COSMOS standard provides a clear technical guide ( COSMOS, 2021). Genetically modified plants are not authorized, but ingredients produced using GMO-derived enzymes can be approved if they comply with the following conditions:

- The enzymes from GMOs are purified before use
- The GMOs are used in closed tanks
- The GMOs are deactivated after the process
- A risk assessment linked to the impact of the release of GMOs into the environment is carried out
- A risk plan to deal with accidental release of GMOs into the environment is established
- A negative PCR or other method must be provided to prove that no DNA from the GMO is present in the final material

Given the expanding role of GMO-derived enzymes in the food and pharmaceutical industries ( Trono, 2019), it is likely that cosmetic ingredients produced using such enzymes could soon be brought to market. In addition, fermentation media must comply with COSMOS standard criteria stating that each ingredient in the medium must be of mineral, vegetable, microbial, animal, or marine origin and, where applicable, must be guaranteed of non-GMO origin.

The IECIC2021 list already contains cosmetic ingredients that are produced by fermentation using GMO microbes, including resveratrol, kojic acid, and hyaluronic acid ( Gomes *et al.,* 2020). These were introduced to the Chinese market before 2020 and are therefore approved as “historic ingredients” both in terms of the chemical entities themselves and the biotechnological processes used to produce them. The IECIC2021 list also contains many other pure compounds of natural plant origin that could be produced by GMO microbes in the future, including ferulic acid (no. 11020), carvacrol (no. 07021), gallic acid (no. 01212), phytic acid (no. 03146) and coumarin (no. 06985). Because these products will be introduced to the Chinese market after 2020, they will require a low-risk approval route. The use of optimized biotechnological processes to manufacture such products could help to reduce the carbon footprint of the cosmetic industry and free up agricultural lands for crops, which would be in keeping with green chemistry principles ( Yi & Ng, 2023).

## *InnCoCells* – innovative high-value cosmetic products from plants and plant cells

**Figure 1.**
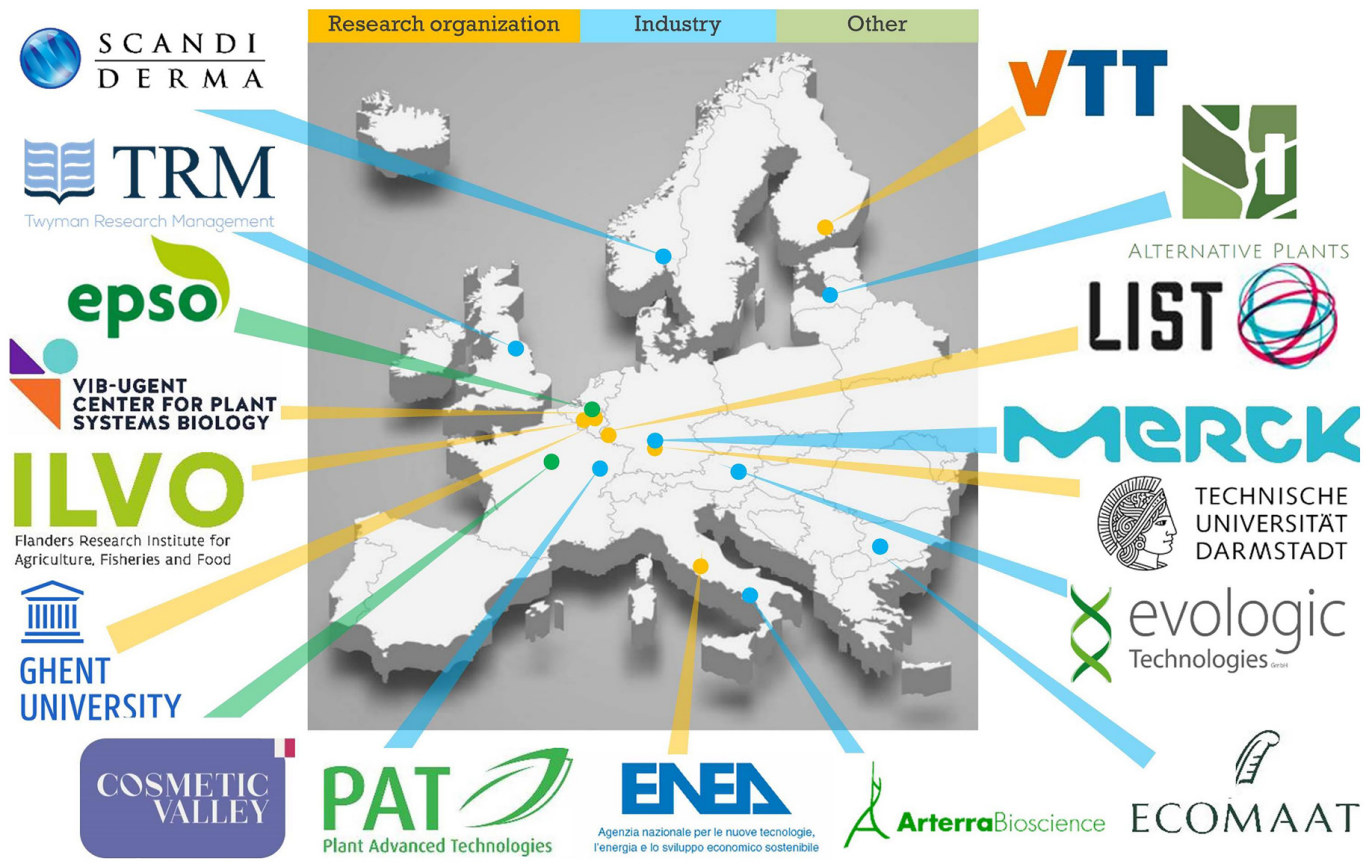
The *InnCoCells* consortium.

**Figure 2.**
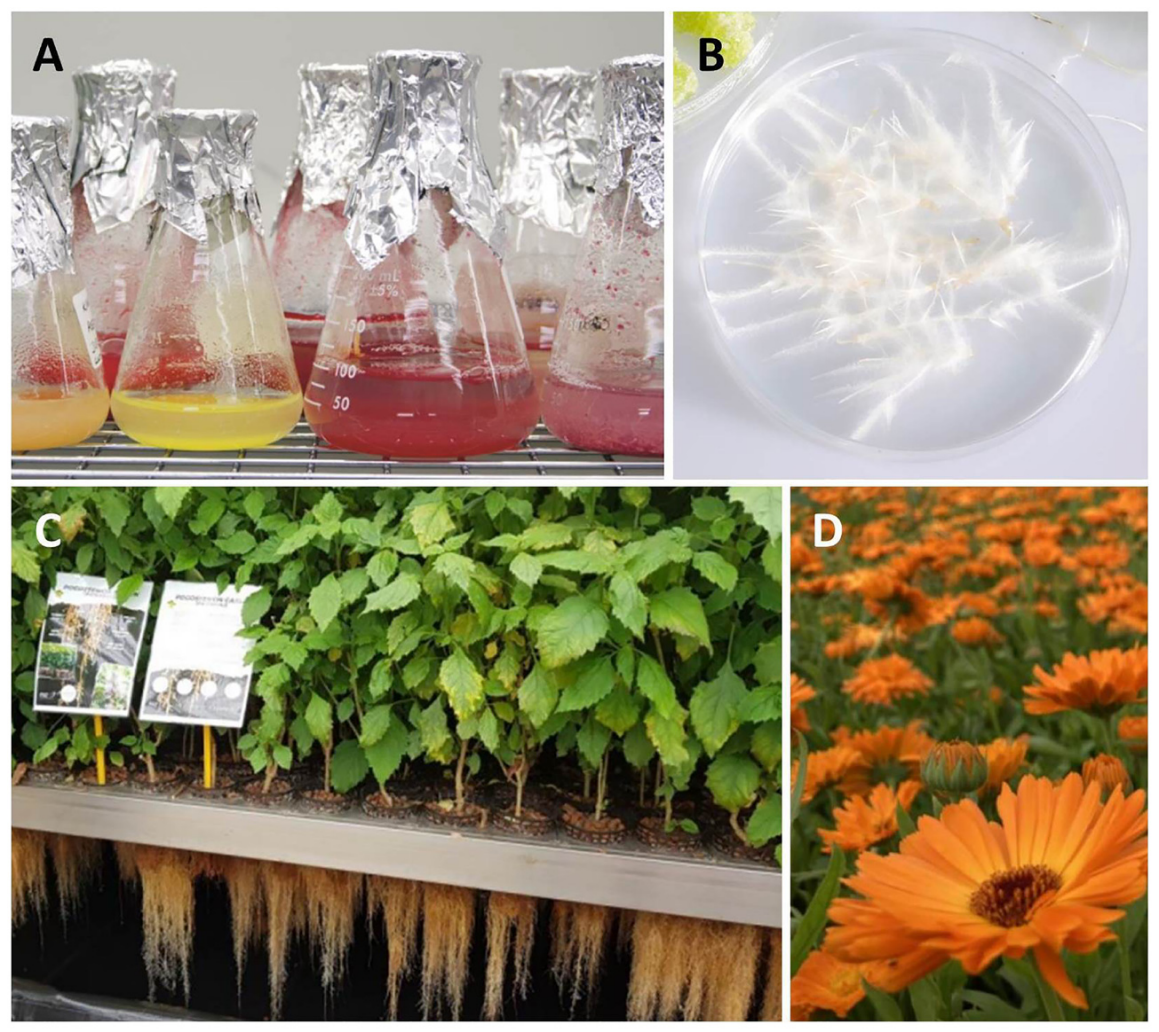
The production systems used in the *InnCoCells* project: ( **A**) Plant cell suspension cultures; ( **B**) Hairy roots; ( **C**) Aeroponic systems; and ( **D**) Field cultivation. Image credits: (A, B) VTT, (C) Plant Advanced Technologies, (D) EV ILVO.

The issues outlined above concerning the role of biotechnology in the development of sustainable, plant-derived cosmetic ingredients led to the conception of *InnCoCells*, a Horizon 2020 project focusing on innovations in bioprospecting, production systems, purification methods and functional bioassays relevant to today’s cosmetics market. The *InnCoCells* consortium consists of 17 partners from 11 countries representing European academic and industrial leaders, and it is coordinated by VTT Technical Research Centre of Finland Ltd ( Figure 1). The project was launched in May 2021 with a budget of €7.9 million and will run until October 2025. The main objective is to bring at least 10 novel, scientifically validated and well-characterized ingredients to the pre-commercial stage. The production systems considered by the project are plant cell suspension cultures, hairy roots, aeroponic systems and greenhouse/field cultivation ( Figure 2). The project has also developed highly innovative methods for the preparation of extracts, detailed metabolic analysis, and the testing of bioactivities to provide scientific evidence of efficacy.

**Figure 3.**
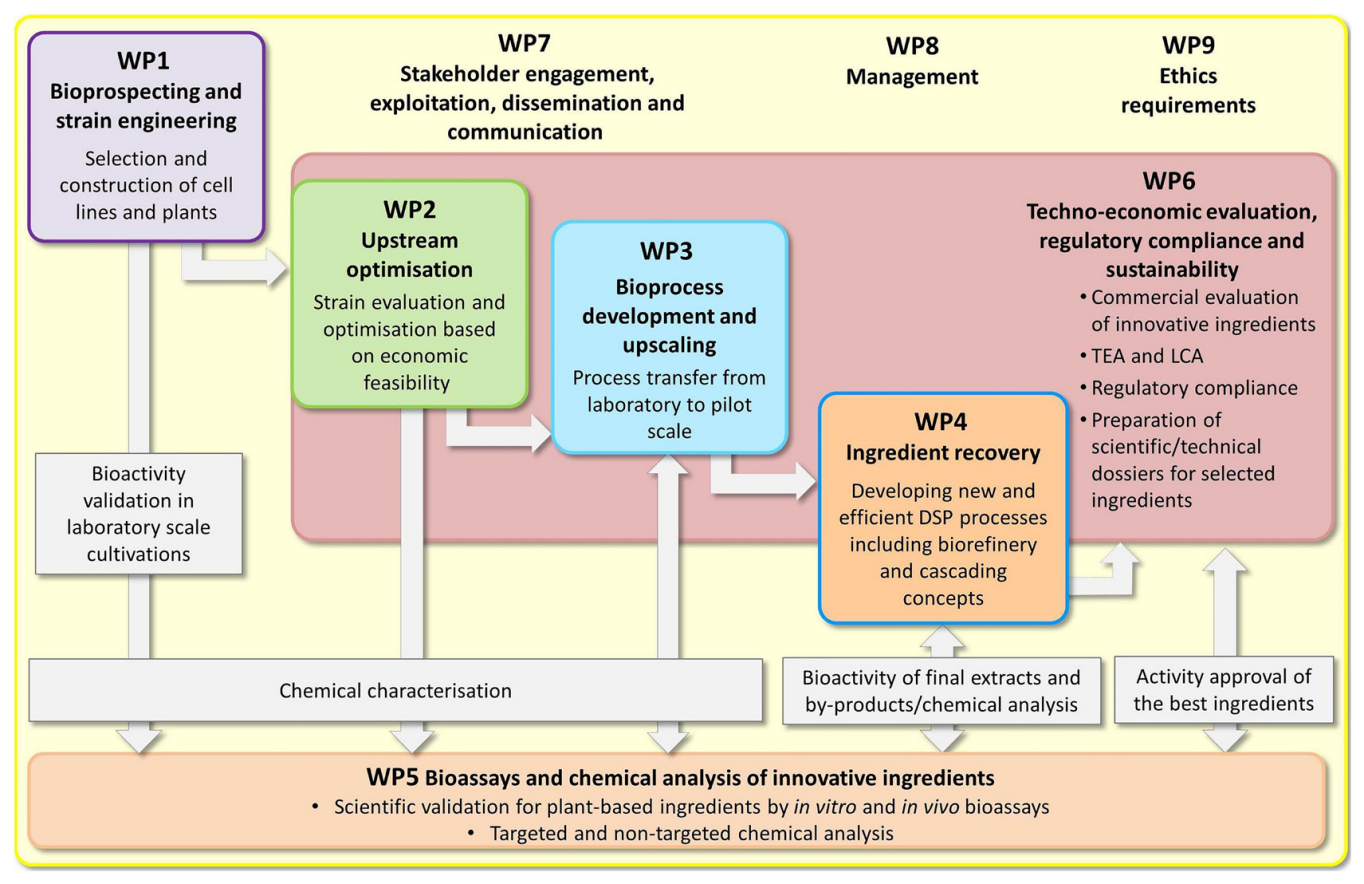
The organization of the *InnCoCells* project.

The work is divided into five scientific work packages ( Figure 3). The bioprospecting phase of the project ( **WP1**) resulted in the identification of ~100 underutilized plant species, by-products or waste fractions from the agrifood industry for further investigation. Based on earlier studies by the partners, preliminary tests and information from the literature, we were able to define 25–30 plant species as the most promising resources. Importantly, all the plant species and extracts were included in the IECIC list and their utilization did not violate international regulations defining access to genetic resources, including the Nagoya protocol as described above. This early work therefore ensured that any ingredients delivered by the project would meet current regulatory standards, making them suitable for commercialization.

We developed tailored cultivation processes in all the *InnCoCells* production platforms to optimize biomass production and the accumulation of specific bioactive metabolites in **WP2**. This provided a panel of candidates (product and process combinations) that were tested for scalability in **WP3**. Accordingly, we scaled four cell culture and hairy root lines up to 300 L and two to 1000 L (defined as pilot scale). For the aeroponic cultivation system, three plant species were scaled up to 100 m ^2^ surfaces (also defined as pilot scale). We developed novel downstream processes based on the pre-treatment of plant biomass using methods such as pulsed electric fields to achieve high yields of extracts with the desired biomolecular composition in **WP4**. The reproducibility of the upstream and downstream processes and the stability of the resulting extracts are important parameters that we are still investigating.

More than 100 hydrophilic and lipophilic extracts have been tested in **WP5** using a range of advanced bioactivity assays, including tests for cytotoxicity, as well as anti-inflammatory, anti-aging, antioxidant and antimicrobial activities, all of which are important in the cosmetics industry, especially in skincare products. Currently, the 25 best-performing ingredients are being tested in four different ex vivo assays based on skin biopsy samples. The composition of extracts with the most promising bioactivities has been assessed in detail by advanced targeted and untargeted metabolomics.

As more ingredients come through the pipeline, we are focusing on regulatory compliance, life cycle assessment and the evaluation of product/process sustainability in **WP6**. This requires extensive documentation and the assessment of techno-economic viability. Regulatory compliance has already been evaluated for a small number of our most advanced ingredients, and we are working on the preparation of safety and technical data sheets, scientific dossiers, and the pre-commercial evaluation of efficacy claims. This work is complemented by an extensive panel of dissemination, exploitation and communication activities in **WP7**. These include an informative website, an active social media presence, the publication of research articles, presentations at scientific conferences, and the organization of public webinars and stands at high-profile cosmetic industry exhibitions such as the annual Cosmetic360 event in Paris. Our Stakeholder Group, made up of cosmetic industry representatives, farmers, academic researchers and cosmetic end-user groups, has guided the selection of promising cosmetic ingredients and has helped to raise awareness about the project by attending our meetings and media events, and by publicizing our brochures, podcasts, and promotional videos. In this way, the *InnCoCells* project aims to span the value chain from discovery, through product and process development and testing, up to the pre-commercial stage, where our industrial partners and Stakeholder Group members have the opportunity to take the resulting ingredients to the next stage on the road to the cosmetics market.

## Outlook

In conclusion, neither market regulations nor the ban on GMO-derived ingredients constitutes an insurmountable hurdle to the use of biotechnology for the production of cosmetic ingredients. We already have clear requirements from national authorities and non-governmental standards to help us define innovative cosmetic ingredients based on biotechnological processes that meet market expectations in terms of safety, validated efficacy and low environmental impact. This is an ideal foundation for *InnCoCells*, the first and currently only large EU-funded public project that is dedicated to the development of sustainable plant-derived cosmetic ingredients with scientifically proven effects. We have already achieved the major project objective, which is to establish environmentally sustainable pilot-scale production and purification technologies for at least 10 active, fully-characterized, pre-commercial cosmetic ingredients. These have been narrowed down from a much broader panel, which has resulted in the bioprospection of 90 different plant items (botanical species and parts thereof), the biodiscovery and sustainable exploitation of at least 10 relevant metabolic pathways in various plant species, the development of a multi-step evaluation pipeline for the testing of plant-derived bioactive molecules and extracts yielding at least 50 scientifically verified active ingredients for cosmetic products, and the optimization of production processes and technologies for at least 20 ingredients based on plant cells/hairy roots, aeroponics, and greenhouse/field cultivation. We have also explored more than 10 agrifood by-products and waste fractions in a cascade biorefinery approach to generate value-added extracts, including olive pomace and ginger press cake. The knowledge and intellectual property accumulated by *InnCoCells* will long outlast the project itself and will form the basis of a new generation of plant-based cosmetic ingredients for the well-informed consumers of tomorrow. 

## Data Availability

No data are associated with this article.
