# Supplementary material for: From plant genetic resources to cosmetic active ingredients: when science meets regulation and market rules
Source: Open Res Eur. 2025 Sep 4;5:165. Originally published 2025 Jun 18. [Version 2] doi: 10.12688/openreseurope.20113.2 (PMC12495392; doi:10.12688/openreseurope.20113.2)
Supplement: Peer review history

# Reviewer report: Reviewer response for version 2

**DOI:** 10.21956/openreseurope.23080.r59606

**Published:** 2025-10-03

**Authors:** Niharika Sahoo Bhattacharya

**Affiliations:** 1 Indian Institute of Technology Kharagpur, Kharagpur, West Bengal, India

The authors have adequately addressed my previous comments. I have no further comments to make.

Where applicable, are recommendations and next steps explained clearly for others to follow? (Please consider whether others in the research community would be able to implement guidelines or recommendations and/or constructively engage in the debate)

No

Does the article adequately reference differing views and opinions?

Partly

Are all factual statements correct, and are statements and arguments made adequately supported by citations?

Yes

Is the rationale for the Open Letter provided in sufficient detail? (Please consider whether existing challenges in the field are outlined clearly and whether the purpose of the letter is explained)

Partly

Is the Open Letter written in accessible language? (Please consider whether all subject-specific terms, concepts and abbreviations are explained)

Yes

Reviewer Expertise:

Drugs and cosmetic regulation, Intellectual Property Laws

I confirm that I have read this submission and believe that I have an appropriate level of expertise to confirm that it is of an acceptable scientific standard.

# Reviewer report: Reviewer response for version 1

**DOI:** 10.21956/openreseurope.21757.r56198

**Published:** 2025-08-23

**Authors:** Niharika Sahoo Bhattacharya

**Affiliations:** 1 Indian Institute of Technology Kharagpur, Kharagpur, West Bengal, India

This Open letter provides an interesting interdisciplinary perspective on biotechnology, plant genetic resources and regulation.  The author deserves commendation for the depth of research conducted on the intersection of biodiversity, cosmetic ingredients, and cosmetics regulation.

 However, a few areas in the article could benefit from further elaboration.

 1.Nagoya protocol to CBD is an important instrument for the sharing of genetic resources and access benefit sharing. However, as the authors pointed out, national legislations are highly diverse when it comes to compliance obligations towards the country providing genetic resources and the national obligations of the receiving country. I feel this section can be elaborated at least with respect to European Union to give a holistic view of diverse national laws or the need of harmonization. Particularly, it will be interesting to learn the role of EU Access and Benefit Sharing (ABS) Regulation (Regulation (EU) No 511/2014) and how it tries to balance the inconsistencies. How successful is ABS mechanism when it comes to exchange of plant genetic resources among different continents?

 2. The basis for selecting certain jurisdictions for comparison remains unclear. On what criteria were the specific national regulatory frameworks chosen, and how do these selections reflect broader trends in global cosmetic regulation? While many Asian countries are known for traditional herbal based cosmetics, authors have only reviewed the China and provided information on permitted plant and new plant ingredients for cosmetics and corresponding regulation. It will be interesting to see the position of other Asian countries as well. For example, country like India which has a rich traditional herbal-based cosmetics and many companies are making its global presence by bringing innovative herbal products. How the newer innovations through biotechnology or other processes are addressed in those countries’ regulations?  

 3. The author rightly highlights the shortcomings and inconsistencies in risk assessment practices across jurisdictions. Yet the discussion would have been stronger had it included a comparative analysis of the registration process for new cosmetic ingredients (NCIs) in the EU alongside China. This would help to understand the regulatory imbalance and potential barriers to development.

 4.This manuscript provides a comprehensive overview of InnCoCell project, which seems to be an essential endeavor from scientist community towards development of new plant based cosmetic ingredients. Such projects highlight the need of bioprospecting and appropriate regulation for the sustainable development of communities as well as biodiversity preservation. More such projects should be promoted in different Countries to advance these crucial objectives.

 5.While it is true that the EU offers well-defined national and central regulatory frameworks, along with clear non-governmental standards, cosmetic regulations globally remain highly inconsistent, with numerous reports highlighting quality and safety concerns. In this context, the authors’ perspective on establishing simple regulatory pathway that ensures both quality and safety while supporting sustainable practices and access benefit sharing will be a valuable addition.

Where applicable, are recommendations and next steps explained clearly for others to follow? (Please consider whether others in the research community would be able to implement guidelines or recommendations and/or constructively engage in the debate)

No

Does the article adequately reference differing views and opinions?

Partly

Are all factual statements correct, and are statements and arguments made adequately supported by citations?

Yes

Is the rationale for the Open Letter provided in sufficient detail? (Please consider whether existing challenges in the field are outlined clearly and whether the purpose of the letter is explained)

Partly

Is the Open Letter written in accessible language? (Please consider whether all subject-specific terms, concepts and abbreviations are explained)

Yes

Reviewer Expertise:

Drugs and cosmetic regulation, Intellectual Property Laws

I confirm that I have read this submission and believe that I have an appropriate level of expertise to confirm that it is of an acceptable scientific standard, however I have significant reservations, as outlined above.

# Response

**Published:** 2025-09-01

**Authors:** Richard Twyman

**Affiliations:** TRM Ltd, UK

Response to reviewer #2 We thank the reviewer for recommending additional content for our article, which has improved its scope and level of detail. We provide a point by point response below.

1. We have added a paragraph to the “use of plant genetic resources section” describing how Regulation (EU) No. 511/2014 applies overarching guidance to the implementation of national ABS regulations in EU member states.
2. In the “approved lists…” section, we have now explained the justification for the selection of Chinese and EU regulatory frameworks, namely that they represent large segments of global cosmetics sales and R&D, and also embody distinct regulatory archetypes (ingredient-centric in China vs product-centric in the EU). In the same section, we have now added a paragraph summarizing the regulatory frameworks in India, Japan, South Korea and ASEAN to provide a broader perspective on how these issues are handled in Asia. We have included, as references, links to documents outlining the primary legislation in these countries.
3. In the same section, we have compared the regulation of cosmetic ingredients in China vs the EU in more detail.
4. We agree that more funding should be available for projects dealing with cosmetic ingredients, and have included a statement at the beginning of the InnCoCells section making this point explicitly.
5. The reviewer raises an excellent point about using this Open Letter as a platform to make recommendations about the establishment of simplified regulatory pathways to harmonize regulatory practice worldwide. We have added such commentary at the beginning of the outlook section and thank the reviewer for this suggestion.

# Reviewer report: Reviewer response for version 1

**DOI:** 10.21956/openreseurope.21757.r56195

**Published:** 2025-08-20

**Authors:** Ahmed Zahidi

**Affiliations:** 1 Mohammed V University, Rabat, Morocco

Title:

 From plant genetic resources to cosmetic active ingredients: when science meets regulation and market rules

 The manuscript addresses a highly relevant and strategic topic at the intersection of plant-based innovation, regulatory complexity, and sustainable cosmetic development. By focusing on the regulatory and biotechnological dimensions of natural ingredient production, and illustrating them through the Horizon 2020 InnCoCells project, the article provides a clear and timely overview that will interest researchers, industry professionals, and policymakers alike.

 The manuscript is scientifically sound and well-structured; however, minor revisions are recommended to improve the precision of certain formulations and to clarify the intended objectives. These improvements will enhance the overall clarity and impact of the article.

 General recommendations:

 *If the journal's editorial guidelines allow, I recommend including a list of abbreviations to improve clarity and reader accessibility.

 Minor corrections

 Abstract:

 I recommend adding one or two key regulatory references in the abstract.

 Please add the term biotechnology in the abstract, especially since it appears in the keywords, to accurately reflect the scientific focus of the article.

 Keywords:

 I recommend citing the following keywords in this order:

 Plant-based ingredients, cosmetics, sustainability, efficacy, regulatory compliance, biotechnology

 The selected keywords accurately reflect the main themes of the article; they are precise, consistent with the manuscript’s content, and enhance its visibility in both scientific and regulatory databases.

 Introduction:

 The introduction provides an overview of current trends in the cosmetics market and the growing interest in natural, plant-based products. However, it relies on a single reference (market statistics) and does not cite any regulatory or technical references, even though these are part of the core of the article. Furthermore, the specific objective of the article is not clearly stated.

 Use of plant genetic resources:

 Please replace:

 “The Rio Convention on Biological Diversity (Secretariat of the Convention on Biological Diversity, 2023) recognizes the right of countries to control access to their own genetic resources (Secretariat of the Convention on Biological Diversity, 2023) and has been ratified by 196 countries.”

 with

 “The Rio Convention on Biological Diversity (Secretariat of the Convention on Biological Diversity, 2023) recognizes the right of countries to control access to their own genetic resources and has been ratified by 196 countries.”

 References:

 Please ensure that all references are formatted according to the journal’s guidelines, with particular attention to the next references, which may contain formatting or content inconsistencies

 Some “Reference Source” entries do not include the date of access, which is generally required for online resources that are subject to updates or changes over time. It is recommended to add the access dates to ensure proper citation standards are met.

 Please replace “BDIH” with “Bundesverband der Industrie- und Handelsunternehmen”

 Please replace “NMPA” with “National Medical Products Administration”

 Please replace

 “Trono D: Recombinant enzymes in the food and pharmaceutical industries. ‎Adv Enz Technol. 2019; 13: 349–87.”

 with

 “Trono D. Recombinant enzymes in the food and pharmaceutical industries. *In *Polaina, J. and MacCabe, A.P. (eds.): Advances in enzyme technology. Amsterdam: Elsevier; 2019: 349–87.”

 My conclusion:

 This manuscript is scientifically sound and has the potential to influence future research. ‎However, minor corrections are required to ensure its clarity and accuracy

Where applicable, are recommendations and next steps explained clearly for others to follow? (Please consider whether others in the research community would be able to implement guidelines or recommendations and/or constructively engage in the debate)

Yes

Does the article adequately reference differing views and opinions?

Yes

Are all factual statements correct, and are statements and arguments made adequately supported by citations?

Partly

Is the rationale for the Open Letter provided in sufficient detail? (Please consider whether existing challenges in the field are outlined clearly and whether the purpose of the letter is explained)

Yes

Is the Open Letter written in accessible language? (Please consider whether all subject-specific terms, concepts and abbreviations are explained)

Yes

Reviewer Expertise:

Pharmaceutical chemistry, Phytochemicals, Nutrition, Iodine deficiency, Public health

I confirm that I have read this submission and believe that I have an appropriate level of expertise to confirm that it is of an acceptable scientific standard.

# Response

**Published:** 2025-09-01

**Authors:** Richard Twyman

**Affiliations:** TRM Ltd, UK

Response to reviewer #1 We thank the reviewer for these helpful comments and suggestions, and provide a point by point response below.

1. The journal’s editorial guide does not allow references to be cited in the abstract and does not, as far as we are aware, include keywords that can be selected by the authors. Although the word “biotechnology” is not in the abstract, the word “biotechnological” is already there. Accordingly, we have not modified the abstract. However, we appreciate the reviewer’s suggestions and have instead cited new references about the regulation of cosmetics in the introduction, and have added the term “biotechnology-based” there too.
2. In the introduction, we have added two recent references about the regulation of cosmetics and have concluded the introduction with a statement outlining the objective of the article.
3. We have removed the repeated citation from the section on the use of plant genetic resources as suggested.
4. We have updated the references highlighted by the reviewer and are grateful for the additional information provided. Upon submission, we included the data of last access to online resources (as we have for the new references added in this update) and we have asked the journal editors to ensure the access dates are included.
